# Supplementary material for: Genome-wide SNPs reveal novel genetic relationships among Atlantic cod (Gadus morhua) from the south coast of Newfoundland, Canada (subdivision 3Ps), Northern cod stock complex, and Gulf of St Lawrence
Source: PLoS One. 2025 Mar 14;20(3):e0317768. doi: 10.1371/journal.pone.0317768 (PMC11908700; doi:10.1371/journal.pone.0317768)
Supplement: S2 Table [file pone.0317768.s002.pdf]

**Table S2: Filtering information for each dataset.**

| **Step** | **Loci or Individuals Retained** |
|---|---|
| FIS<-0.3<br>Heterozygosity>0.6 | Kept 679719 sites<br>Kept 378/378 individuals |
| Minor Allele Frequency (maf) 0.05 | Kept 56260/679719 sites<br>Kept 378/378 individuals |
| Minimum Read Depth (minDP) 5 | |
| Maximum Number of Alleles (max-alleles 2) | |
| Maximum Missing Data (max-missing 0.7) | |
| Maximum Missing Data by Individual (max-missing 0.3) | Kept 56260/56260 sites<br>Kept 326/378 individuals |
| Related Individuals 0.5 | Kept 56260/56260 sites<br>Kept 324/326 individuals |
| Non-Nuclear Loci Removal | Kept 55675/56260 sites<br>Kept 324/324 individuals |
| **Remove individuals from 2005 (wrong year)** | Kept 319/324 individuals |
| **Linkage Equilibrium (--min-r2 0.2; NEUTRAL ONLY)** | Kept 40176/55675 sites<br>Kept 319/319individuals |
| **Linkage Group Removal (NEUTRAL ONLY)** | 2044 loci removed |
| **Outliers (NEUTRAL ONLY)** | 25 loci removed |
| **FINAL DATASETS** | **FULL: 55675 sites, 319 individuals** |
| | **NEUTRAL: 38111 sites, 319 individuals** |
