# Supplementary figures and images for: Genome-wide SNPs reveal novel genetic relationships among Atlantic cod (Gadus morhua) from the south coast of Newfoundland, Canada (subdivision 3Ps), Northern cod stock complex, and Gulf of St Lawrence
Source: PLoS One. 2025 Mar 14;20(3):e0317768. doi: 10.1371/journal.pone.0317768 (PMC11908700; doi:10.1371/journal.pone.0317768)

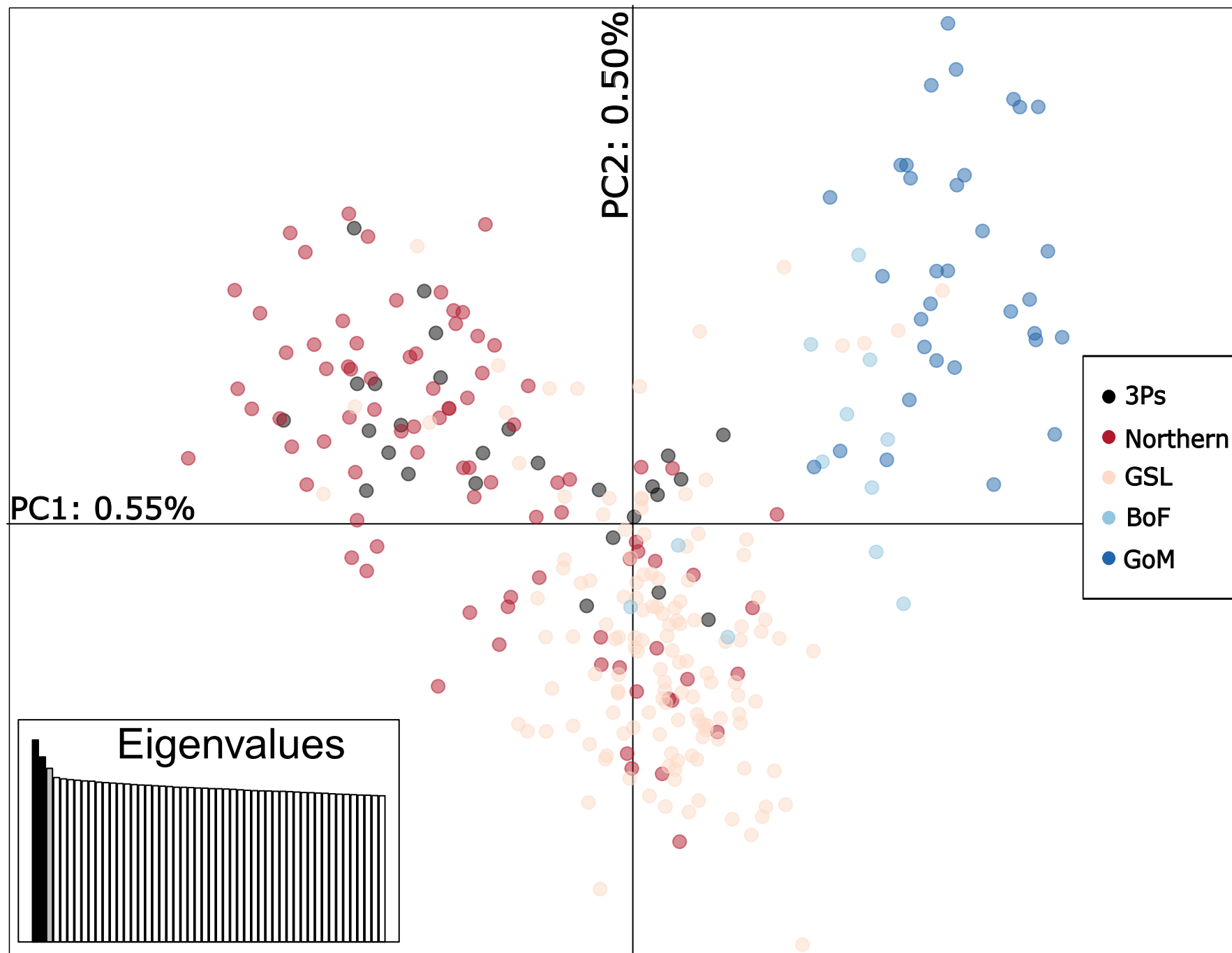

Supplement: S1 Fig — Detection methods include BayeScan, PCAdapt with Bonferroni correction, and PCAdapt with Benjamini-Hochberg correction. Consensus outliers remained the same with addition of third outlier detection method (25 consensus). Northern = Northern cod stock (2J3KL), GSL = Gulf of St. Lawrence (4RST), BoF = Bay of Fundy (4X), GoM = Gulf of Maine (5Y). [file pone.0317768.s005.pdf]

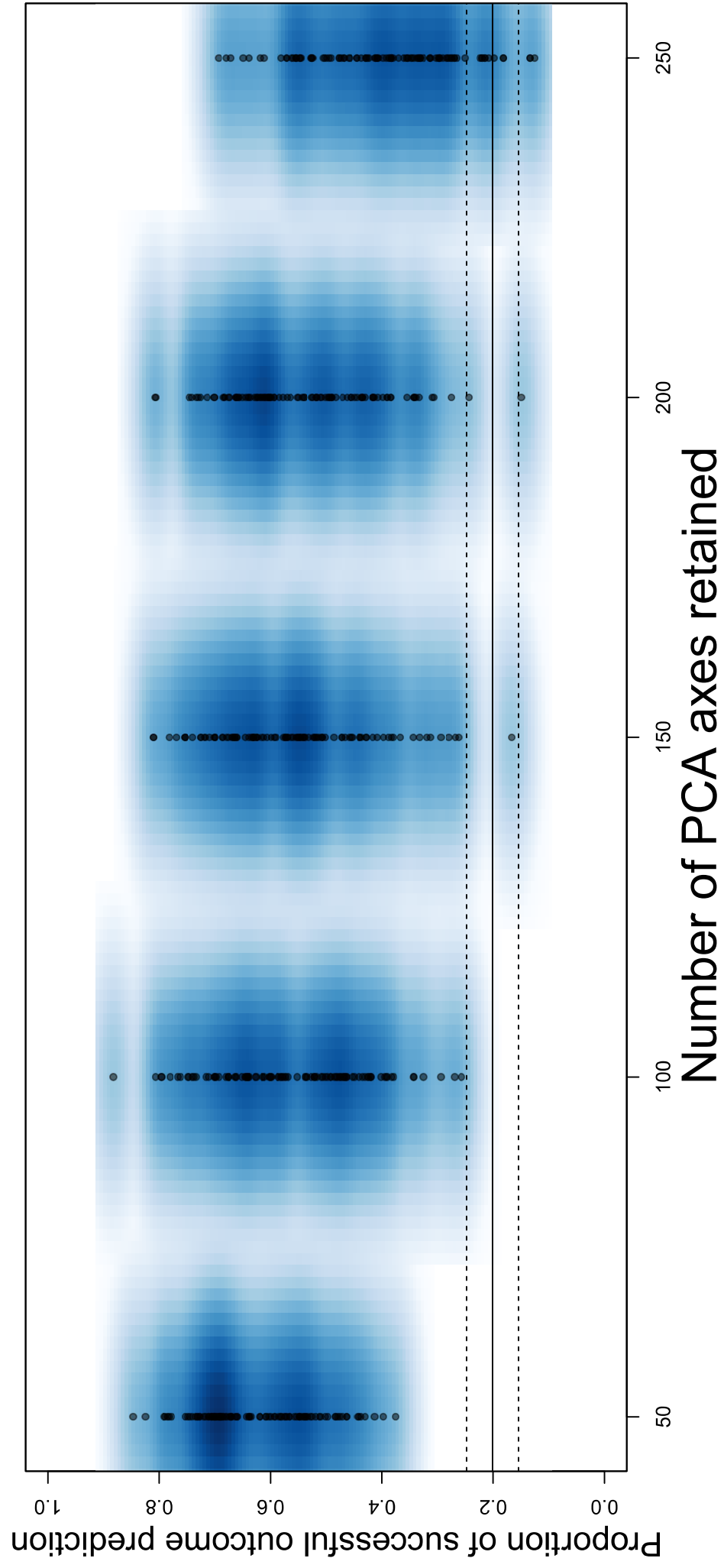

Supplement: S2 Fig — Northern = Northern cod stock (2J3KL), GSL = Gulf of St. Lawrence (4RST), BoF = Bay of Fundy (4X), GoM = Gulf of Maine (5Y). [file pone.0317768.s006.pdf]

PC1: 2.84%

PC2: 1.32%

Eigenvalues

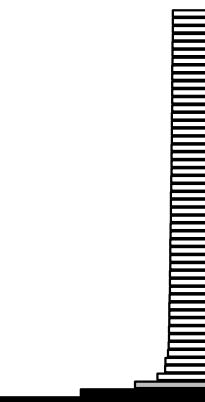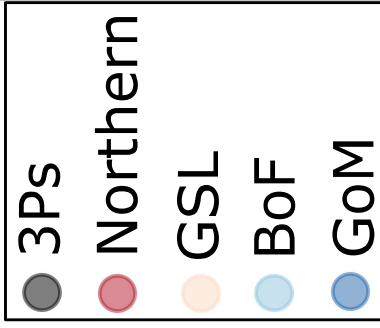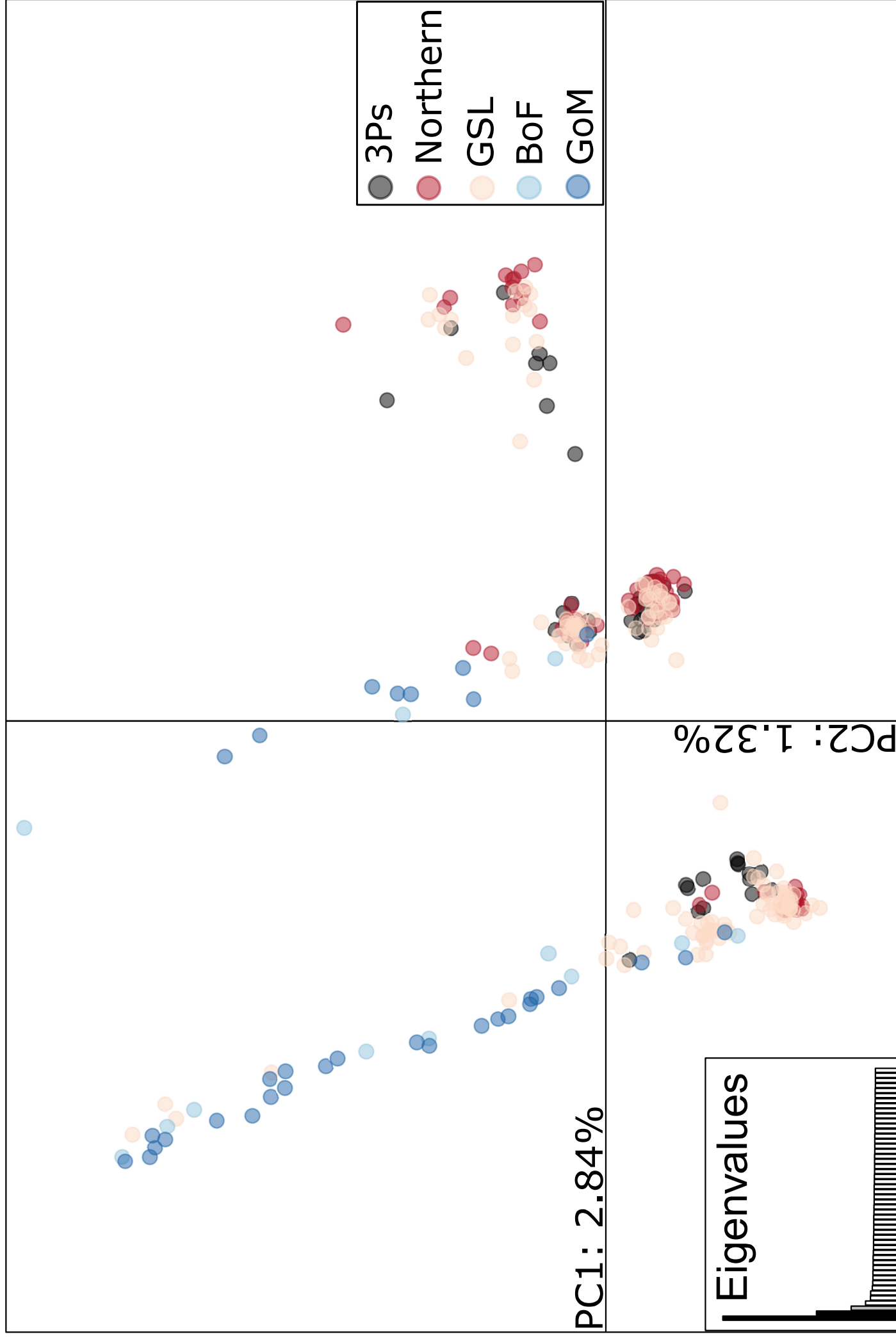

Supplement: S3 Fig — Lowest root mean squared error and highest proportion of successful outcome prediction indicates 50PCs (RMSE of 0.393). [file pone.0317768.s007.pdf]

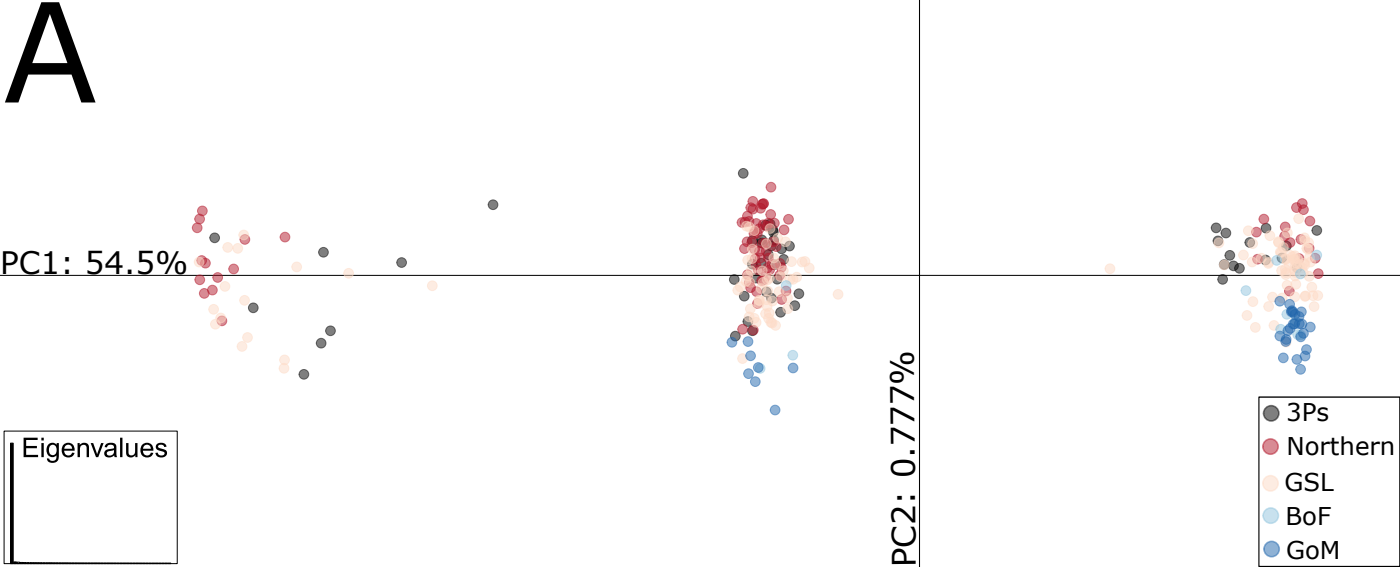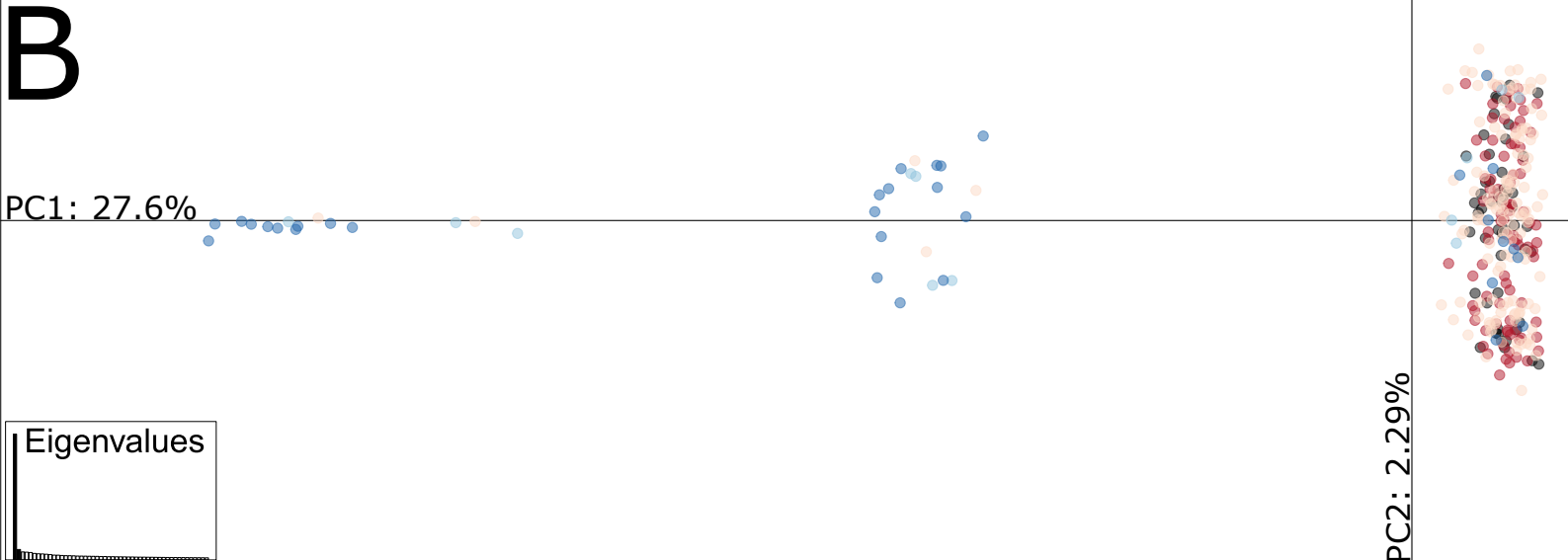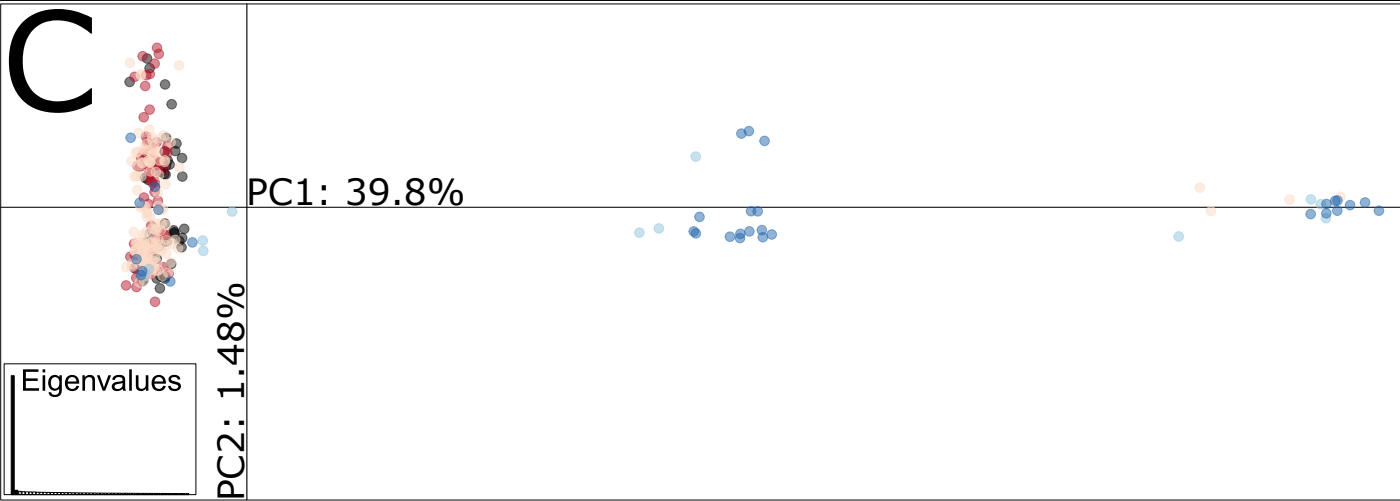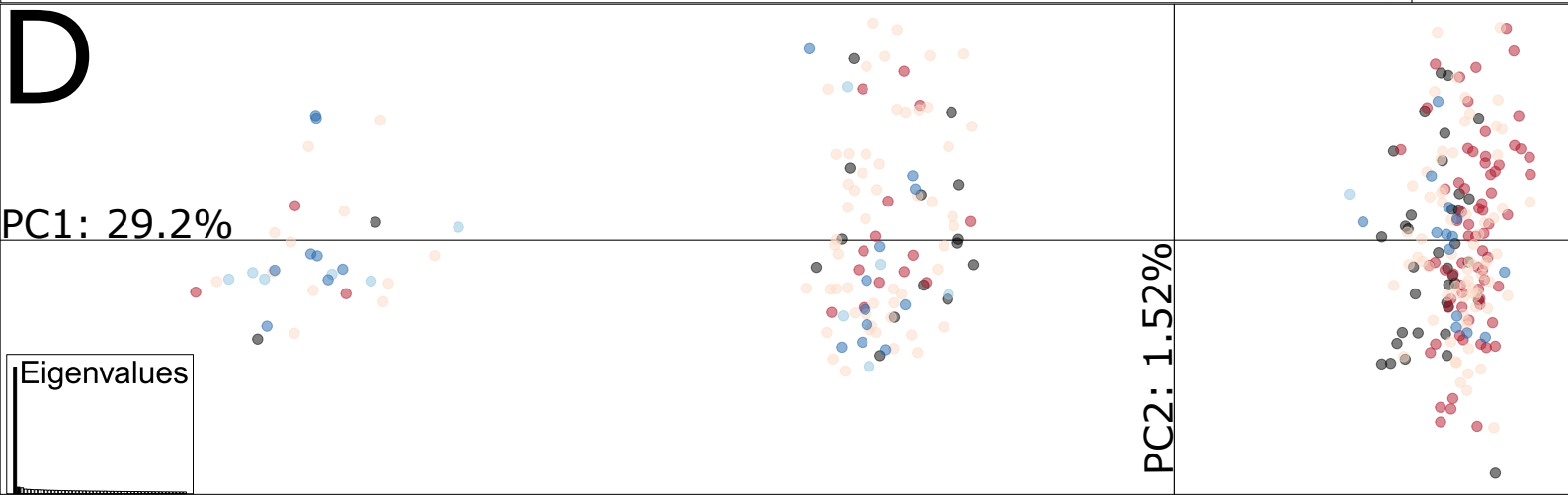

Supplement: S4 Fig — Three groups are shown along the PC1 axis, corresponding to genotypes from LG01. There is some separation of southern populations along the y-axis (Gulf of Maine, Bay of Fundy) from the from the other populations (Northern, Gulf of St. Lawrence, 3Ps). Northern = Northern cod stock (2J3KL), GSL = Gulf of St. Lawrence (4RST), BoF = Bay of Fundy (4X), GoM = Gulf of Maine (5Y). [file pone.0317768.s008.pdf]

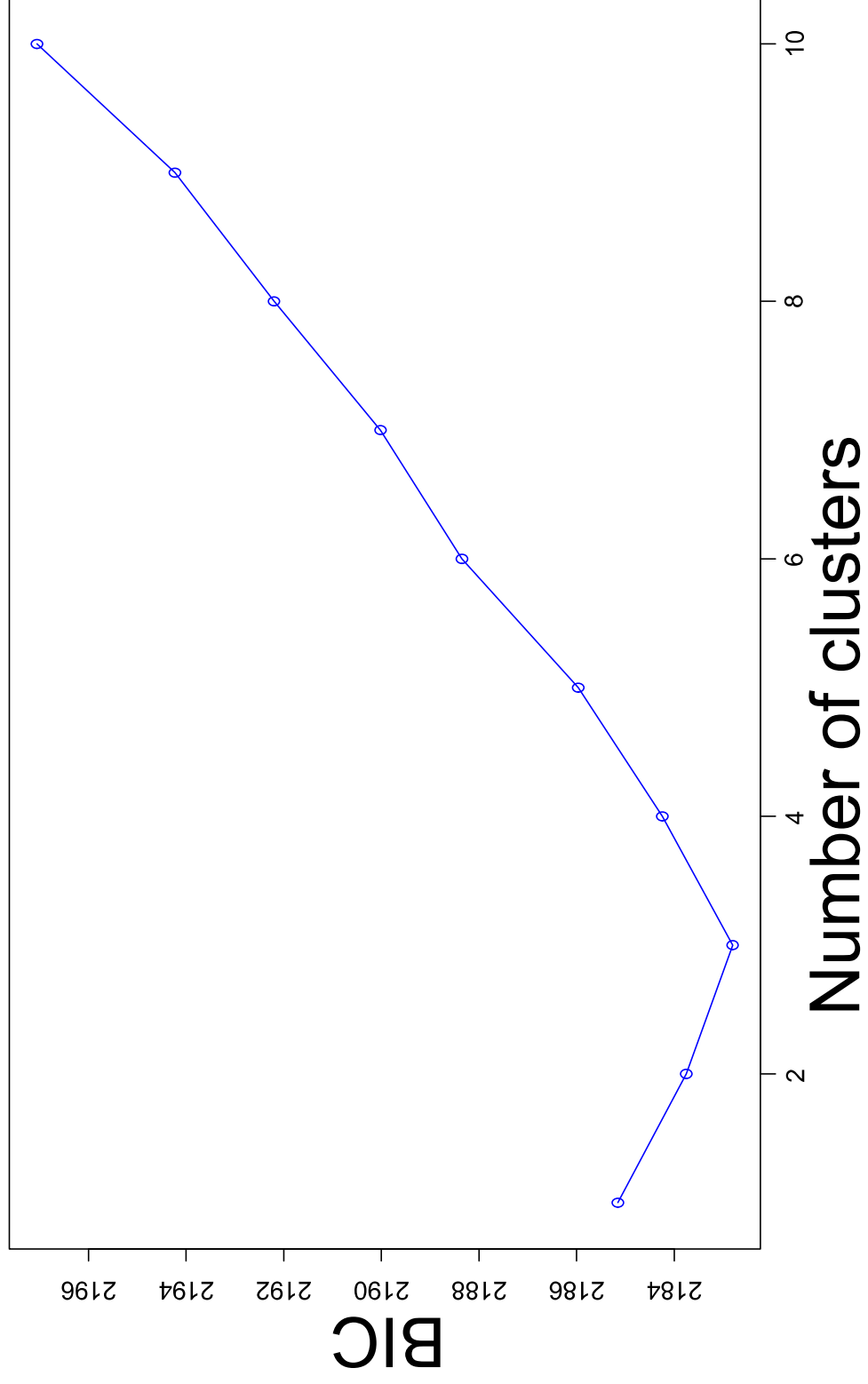

Supplement: S5 Fig — Location of linkage groups were determined using the Manhattan plot on the full dataset (see Fig 2). Three clusters are seen in each plot, corresponding to the three possible genotypes. A) Linkage group 1 (LG01; 1892 SNPs). B) Linkage group 2 (LG02; 563 SNPs). C) Linkage group 7 (LG07; 1027 SNPs). D) Linkage group 12 (LG12; 1017 SNPs). [file pone.0317768.s009.pdf]

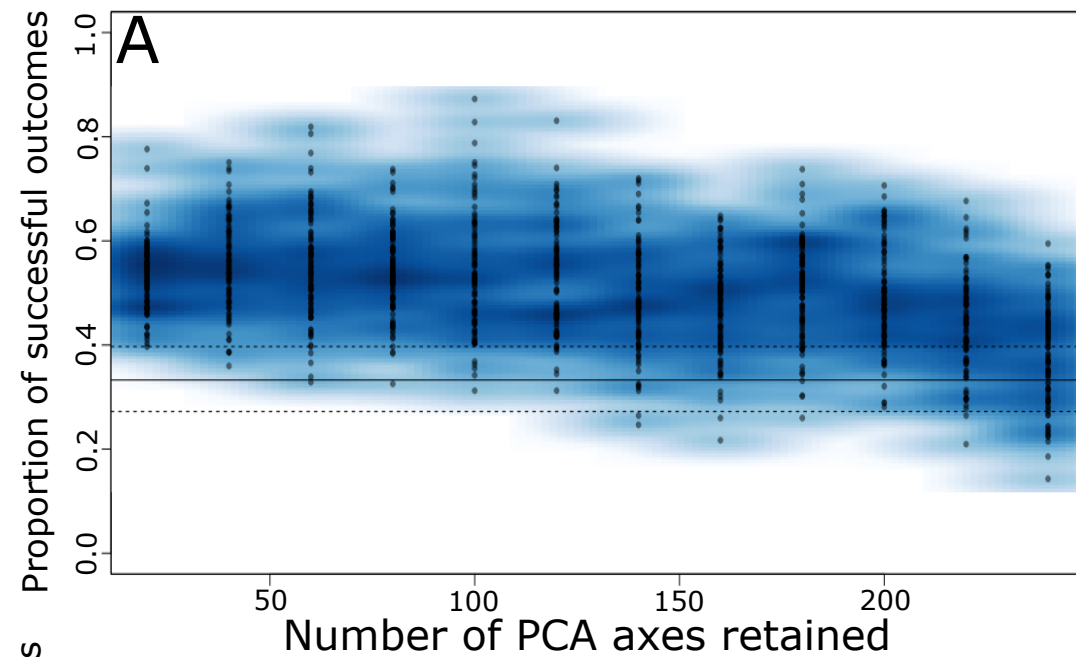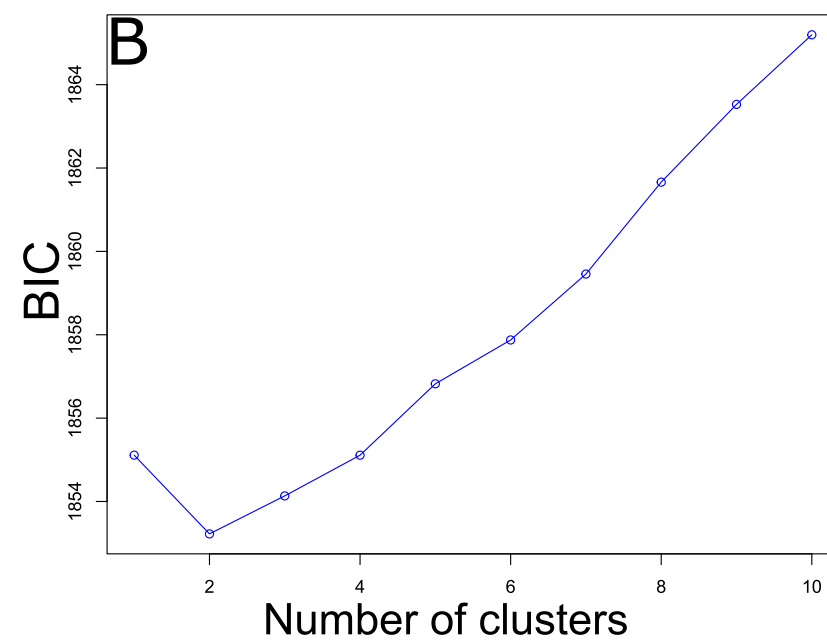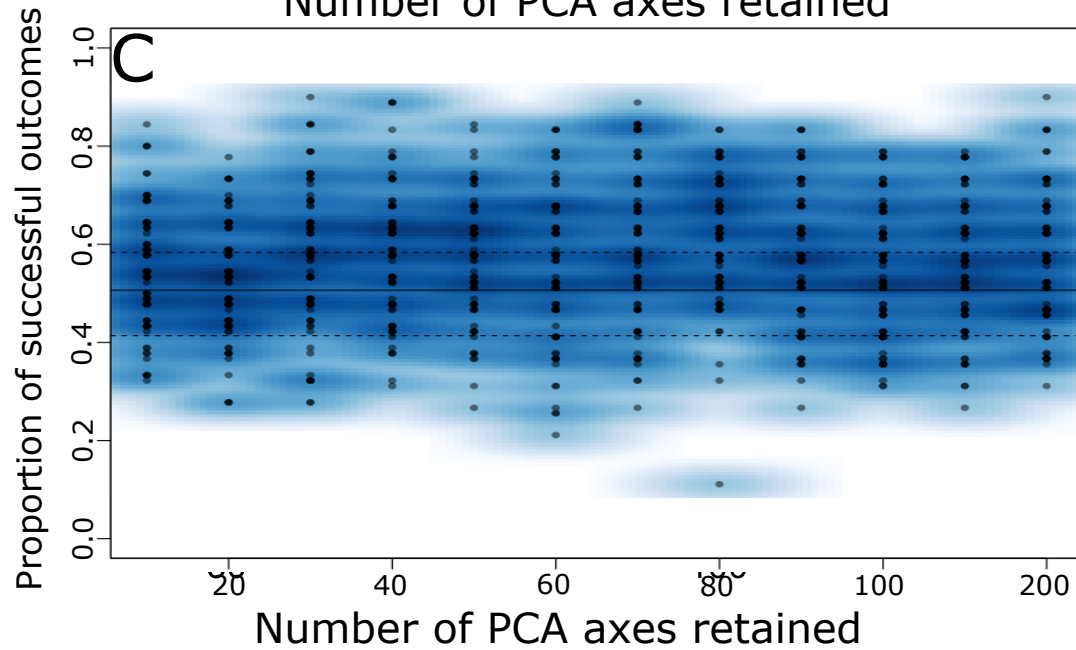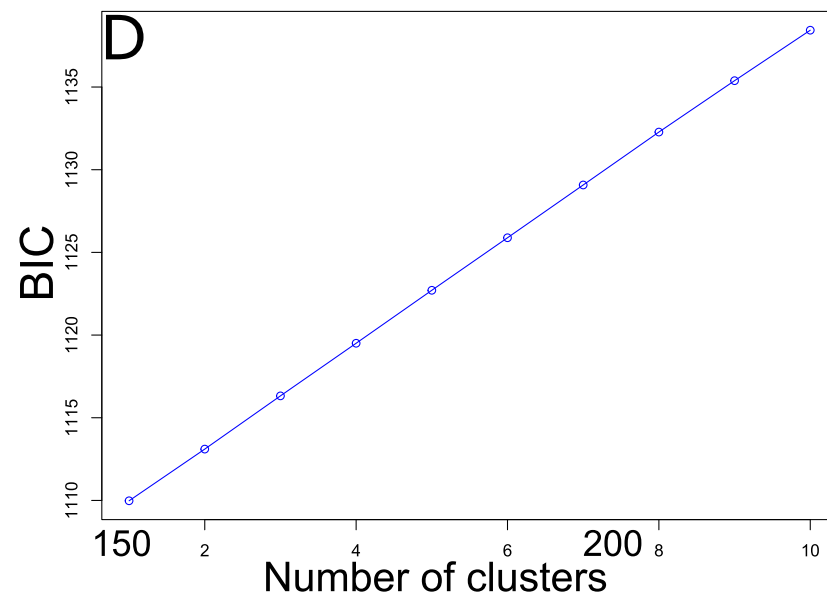

Supplement: S6 Fig — BIC plot with 50PCs retained (as indicated by DAPC cross-validation) BIC plot shows an indication for 3 clusters. [file pone.0317768.s010.pdf]

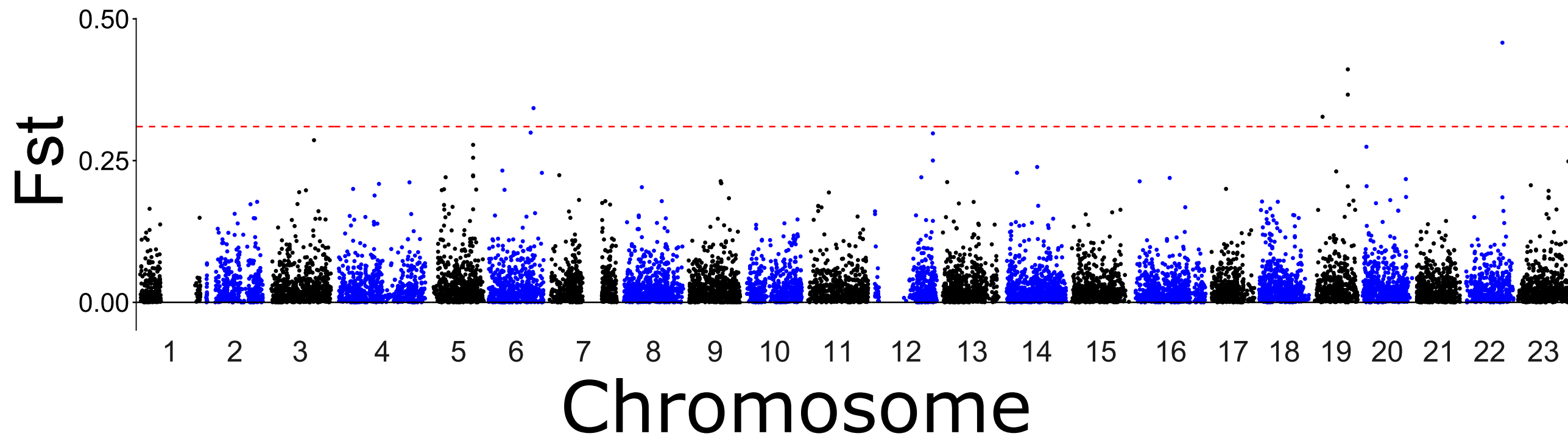

Supplement: S7 Fig — A) Including Gulf of St. Lawrence, individuals from Bay of Fundy and Gulf of Maine removed. B) Individuals from Gulf of St. Lawrence, Bay of Fundy and Gulf of Maine removed. BIC plot with 40PCs retained (RMSE of 0.453) C) Neutral dataset including Gulf of St. Lawrence, individuals from Bay of Fundy and Gulf of Maine removed. D) Individuals from Gulf of St. Lawrence, Bay of Fundy and Gulf of Maine removed, 80PCs retained. This is the number of PCs indicated by DAPC cross-validation (RMSE of 0.401). Dip at 2 clusters is seen in only B. [file pone.0317768.s011.pdf]
