# Supplementary material for: Genome-wide SNPs reveal novel genetic relationships among Atlantic cod (Gadus morhua) from the south coast of Newfoundland, Canada (subdivision 3Ps), Northern cod stock complex, and Gulf of St Lawrence
Source: PLoS One. 2025 Mar 14;20(3):e0317768. doi: 10.1371/journal.pone.0317768 (PMC11908700; doi:10.1371/journal.pone.0317768)
Supplement: Attachment — Submitted filename: Response to Reviewers.docx [file pone.0317768.s013.docx]

**Academic Editor:**

*1. Please ensure that your manuscript meets PLOS ONE's style requirements, including those for file naming. The PLOS ONE style templates can be found at https://journals.plos.org/plosone/s/file?id=wjVg/PLOSOne_formatting_sample_main_body.pdf and https://journals.plos.org/plosone/s/file? id=ba62/PLOSOne_formatting_sample_title_authors_affiliations.pdf*

>>> The manuscript has been formatted to fit PLOS ONE’s requirements, including all figures.

*2. Thank you for stating the following financial disclosure: "Fisheries and Oceans Canada Competitive Science Research Fund grant #2021-22_FS-14_NL to DV Natural Sciences and Engineering Research Council of Canada (NSERC) Discovery Grant to CCD and SAP (RGPIN-2020–04112 & RGPIN-2023-04132, respectively) New Brunswick Innovation Foundation STEM and Social Innovation award to SB"*

*Please state what role the funders took in the study. If the funders had no role, please state: ""The funders had no role in study design, data collection and analysis, decision to publish, or preparation of the manuscript."" If this statement is not correct you must amend it as needed. Please include this amended Role of Funder statement in your cover letter; we will change the online submission form on your behalf.*

>>>We have included a funding statement in the updated cover letter of this manuscript.

*3. Thank you for stating the following in the Acknowledgments Section of your manuscript: "This work was funded by Fisheries and Oceans Canada Competitive Science Research Fund grant #2021- 22_FS-14_NL to DV and Natural Sciences and Engineering Research Council of Canada (NSERC) Discovery Grant to CCD and SAP (RGPIN-2020–04112 & RGPIN-2023-04132, respectively). We thank Fisheries and Oceans Canada technicians for collecting scale samples and Genome Quebec for sequencing. S.B. was supported by a New Brunswick Innovation Foundation STEM and Social Innovation award. We also thank Remy Rochette and Stephen Carr for feedback during SB’s MSc thesis defence." We note that you have provided funding information that is not currently declared in your Funding Statement. However, funding information should not appear in the Acknowledgments section or other areas of your manuscript. We will only publish funding information present in the Funding Statement section of the online submission form. Please remove any funding-related text from the manuscript and let us know how you would like to update your Funding Statement. Currently, your Funding Statement reads as follows: "Fisheries and Oceans Canada Competitive Science Research Fund grant #2021-22_FS-14_NL to DV Natural Sciences and Engineering Research Council of Canada (NSERC) Discovery Grant to CCD and SAP (RGPIN-2020–04112 & RGPIN-2023-04132, respectively) New Brunswick Innovation Foundation STEM and Social Innovation award to SB" Please include your amended statements within your cover letter; we will change the online submission form on your behalf.*

>>> We have removed the funding information from the Acknowledgments section of the manuscript and added a thank you for the anonymous reviewers. Our funding statement contains all funding information and does not require an update.

*4. When completing the data availability statement of the submission form, you indicated that you will make your data available on acceptance. We strongly recommend all authors decide on a data sharing plan before acceptance, as the process can be lengthy and hold up publication timelines. Please note that, though access restrictions are acceptable now, your entire data will need to be made freely accessible if your manuscript is accepted for publication. This policy applies to all data except where public deposition would breach compliance with the protocol approved by your research ethics board. If you are unable to adhere to our open data policy, please kindly revise your statement to explain your reasoning and we will seek the editor's input on an exemption. Please be assured that, once you have provided your new statement, the assessment of your exemption will not hold up the peer review process.*

>>>All code and genotype data are now available on the paper’s GitHub repository (<https://github.com/SarahBabaei/3Ps_Atlantic_Cod>). Raw sequencing reads have been uploaded to Sequence Read Archive under BioProject number PRJNA1178142, to be released upon publication.

*5. Please include your full ethics statement in the ‘Methods’ section of your manuscript file. In your statement, please include the full name of the IRB or ethics committee who approved or waived your study, as well as whether or not you obtained informed written or verbal consent. If consent was waived for your study, please include this information in your statement as well.*

>>>We have now included an ethics statement in the Methods section of our manuscript.

L181-187: In Canada, protocols or inclusion in animal use inventories is not required for work involving fishes that are lethally sampled under government or other regulatory mandates for established fish inspection procedures, abundance estimates, and other population parameters required for assessing stocks. As the fish collected for this project were sampled as a part of Fisheries and Oceans Canada’s annual spring multispecies bottom trawl survey which is used to monitor the abundance of fish and shellfish populations in NAFO Divisions 3LNOPs, these samples fell under that exemption.

**Reviewer 1:**

*Reviewer #1: In this manuscript, Babaei et al looked at the population structure of Northern Cod's west Atlantic populations focusing on individuals from the 3Ps NAFO division. The study uses a reduced representation approach and focuses on neutral loci to estimate population structure. Results suggest that cod in the west Atlantic is a metapopulation and the stock from 3Ps is a mix of Northern Cod (2J3KL) and Gulf of St. Lawrence (3Pn4RS) stocks. The paper is clear and the methodology is appropriate, however, there are major points that should be addressed before I can recommend its publication.*

>>> We are glad the reviewer likes the manuscript overall and would like to thank them for the thoughtful comments which have improved the manuscript. In particular, we have clarified the main take-home message, which is that there are two previously undescribed genetic groups. Two have approximately equal proportions in these groups, whereas 4RST is mostly represented by one and 2J is the other.

*- Although, indeed, previous studies haven't looked specifically at 3Ps, there are population genetics studies in cod that include samples from this NAFO division. Although other papers from the same authors have been cited, I am surprised that these studies haven't been mentioned or discussed in the manuscript: Clucas et al., 2019; Kess et al., 2020; Ruzzante et al., 2000; Therkildsen et al., 2013.*

>>> We rephrased the text throughout the manuscript to clarify that while other studies have not focused specifically on 3Ps, several have included samples from 3Ps. We now refer to Ruzzante et al. 2000, Clucas et al. 2019, Kess et al. 2020, and Therkildsen et al. 2013 throughout the manuscript.

*- The analyses are appropriate however, regarding detecting outliers, it is recommended to use at least 3 outliers detection methods as it is well known that different methods detect different sets of outlier SNPs. A more conserved approach would be to consider all SNPs found by BayeScan and PCAdapt as outliers, instead of the common SNPs considering the goal is to have a set of neutral markers. In the manuscript, there is no information on the number of outliers each of the two methods detected, so is unknown the proportion of SNPs that weren’t found in common.*

>>>We agree there are different approaches to identifying and filtering for outliers. Based on your comment, we explored the effect of removing all outliers detected in either method. Their exclusion did not meaningfully change our results, so we left the original analyses in the main text. We also explored a third outlier method (Benjamini-Hochberg) and found that the consensus outliers did not change. When all outliers from all three methods were removed, the results did not substantially change (see figure below). The genepop file with all outliers removed is available on GitHub. Additionally, we now state the number of outliers each method detected in the methods section and added a full list of the outliers to the manuscript’s GitHub repository.

Link to manuscript’s GitHub repository: <https://github.com/SarahBabaei/3Ps_Atlantic_Cod>

Figure: A) PCA from manuscript B) PCA with all outliers removed from all 3 outlier methods

*- To confirm that all SNPs selected were neutral, a HWE test should be performed during the filtering step which is not shown in the methods.*

>>> We note that applying a HWE filter to RADseq data can obscure subtle population structure (see Pearman et al. 2022) and thus we intentionally avoided this filter in the original submission. Deviations from HWE (and reductions in heterozygosity) can be caused by many factors beyond selection, with the primary factor of interest Wahlund effects. We have added this to the methods section.

L223-225: A Hardy-Weinberg Equilibrium (HWE) filter was not included as literature suggests that HWE filters can obscure subtle population structure commonly seen in marine species [53, 54].

*- The paragraphs explaining the changes in PCAs when removing genetic groups can be confusing. I recommend using hierarchical genetic structure plots.*

>>> We substantially revised these paragraphs to better explain the PCA results (L278-283, L289-298). We do conduct a hierarchical analysis insofar as we plot a PCA with (1) all samples (Fig 3A) and (2) the Bay of Fundy and Gulf of Maine removed (Fig 3C). To stay focused on the main results, we removed the supplemental figure that had explored PCAs with various sampling sites removed.

*- I would recommend carefully reading Clucas et al (2019), they too found outliers in chromosomes 18 and 21, which can enrich the discussion section.*

>>> We thank the reviewer for pointing this out. Based on your comment, we have integrated this paper’s results into the discussion section.

L434-438: Clucas et al. [22] also found outliers on chromosomes 18 and 21, including Esr2, when comparing cod from St. Pierre Bank (3Ps) to cod in US waters (western Gulf of Maine winter spawners, Georges Bank, Cox Ledge, eastern Gulf of Maine, and Great South Channel). All other outliers they found also corresponded to genes that play a role in various reproductive functions [22].

*- Do the juveniles cluster differently? Also, it is said that other stocks use the area (3Ps) as spawning ground but not all samples from the other NAFO divisions were sampled during spawning season, this should be discussed.*

>>> Based on your comment, we ran a PCA using only 3Ps juveniles and the genetic clusters show almost identical results to the PCA including all individuals (see figure below). Due to the rest of the populations having only 0-5 juveniles and the low sample size of 3Ps (27 juveniles), this figure was not included in the main text of our manuscript. However, we added this figure has to the supplementary information (S1 Figure) and the corresponding dataset has been uploaded to the manuscript’s GitHub repository. Regarding your second point, we added more text about the seasonality of sampling, and the potential implications on our results, to the Discussion.

L293-294: Results were similar when only juvenile individuals from 3Ps were used (S1 Fig.).

L454-459: We also note that our study contained more 3Ps juveniles and non-spawning adults than spawning adults, samples with a lack of maturity information, as well as samples from different seasons and years. This combination of samples across maturity stages and time limits our ability to confidently determine each individual’s population of origin, as Northwestern Atlantic cod are known to undertake long migrations [65].

Link to GitHub: <https://github.com/SarahBabaei/3Ps_Atlantic_Cod>

Figure: A) PCA from manuscript containing adults and juveniles from 3Ps B) PCA only containing juveniles from 3Ps

Other recommendations:

*- The title is broad, I would advise a more detailed title.*

>>>Thank you for your comment, we changed the title to be more specific.

Title: ‘Genome-wide SNPs reveal novel genetic relationships among Atlantic cod (Gadus morhua) from the south coast of Newfoundland, Canada (subdivision 3Ps), Northern cod stock complex, and Gulf of St Lawrence.’.

*- I recommend being consistent when using location names vs NAFO divisions, for people unfamiliar with the area can be confusing.*

>>>We agree and have made changes throughout the MS to improve consistency in naming.

*- As seasonality is mentioned, it would be good to include in the introduction a brief description of cod’s migratory behavior and spawning time.*

>>>We added a paragraph on cod migratory behaviour and spawning time to the introduction to emphasize the effect seasonality may have on the study.

L129-144: Northwest Atlantic cod also display differences in spawning times and migratory behaviour. Spawning has been observed in waters off the coast of southern Newfoundland (subdivision 3Ps), including in Placentia and Fortune Bays, Burgeo and St. Pierre Banks, and the Halibut Channel [43]. However, cod do not all spawn in the same season, as there is evidence for Northwestern Atlantic cod spawning from winter to autumn, depending on the region [25, 44]. Northern cod stock complex spawn in other areas as well, including the Grand Banks (3L), Belle Isle Banks (3K), and Hamilton Bank (2J) [43-45]. Furthermore, cod are known to undertake long distance migrations [43]. A review of a century of tagging data suggests that individual cod may return to a particular spawning location year after year, a behaviour called ‘homing’ [43]. Migrating cod move through the Northwest Atlantic during different seasons [43]. Tagging and genetic studies have provided strong evidence that individuals from the northern Gulf of St. Lawrence (4RS) stocks and Northern cod stock complex (3KL) stocks migrate to/through southern Newfoundland waters (3Ps). Cod from farther south (e.g. 4VsWX) and trans-Laurentian cod (4TVn), however, were not seen to move north of the Laurentian Channel [31-33] (including into 3Ps). Thus, tagging and genetic evidence suggests that 3Ps may contain a mixed stock rather than a genetically isolated population.

*-There are two paragraphs about the inversions in the intro, as the focus of the study is the neutral markers, I recommend reducing this information and providing more details about the genetic structure in the region found by different markers: microsatellites and SNPs array.*

>>> We shortened the introductory text about inversions into 1 paragraph (L65-83). We also expanded the introductory text on previous genetic structure assessments of the species in the region, e.g. we added details on Ruzzante et al. 2002.

L116-127: For example, Ruzzante et al. [42] used samples from the Gulf of St. Lawrence (4RT), southern Newfoundland (4Vn, 3Pn, 3Ps), and eastern Scotian Shelf (4Vs) to determine their contributions to overwintering Gulf of St. Lawrence cod. Pre-, post-, and spawning cod were genotyped at six microsatellite loci. They found highest contributions from waters off southern Gulf of St. Lawrence (NAFO 4T), southeast and central Newfoundland, Cape Breton Island region, and 3Ps bays (Fortune and Placentia Bays), respectively. Genetic distances among groups were also investigated, with the largest distances being between Gulf of St. Lawrence (NAFO 4RT) and southern Newfoundland waters (NAFO 4Vn, 3Pn, 3Ps). This study suggested that changes in population dynamics and structure could be influenced by seasonal difference in spawning and migration. Genetic differences among fish sampled were marginal, perhaps due to low sample size (for example, only three fish were collected from 3Ps) [42].

Minor comments:

*Line 28: Southern should be capitalized?*

>>>Done.

*Line 30: Not true! although not the main focus different previous studies have included 3Ps samples.*

>>>We agree and have changed the sentence to start with “3Ps has not been the focus of...”

L30-32: However, 3Ps has not been the central focus of previous large-scale genomic analyses of population structure, a knowledge gap that we address using single nucleotide polymorphisms.

*Line 38: "Which defines population structure"? not sure what this is referring to.*

>>>We changed ‘which defines’ to ‘indicating there may be subtle’ to make the sentence clearer.

L37-39: Genetic clustering also suggests that there are two subtle subclusters of Northern stock complex and 3Ps cod, indicating there may be subtle population structure within the Northern stock complex and surrounding zones.

*Line 54: What makes it well-suited for population genomic studies? what species wouldn't be well suited?*

>>>We removed this to avoid confusion and merged the rest of the sentence into the next sentence.

L54-55: Atlantic cod (*Gadus morhua*) is an economically important fish species found in coastal waters on continental shelves throughout the North Atlantic [5].

*Line 61: Not clear the Nov-April?*

>>>We have removed this for clarity

*Line 78: Include reference*

>>>We added references [18, 19]

*Line 120: it is important the sampling of juveniles, spawning and non-spawning adults? nothing in the results or discussion about this.*

>>>We added percentages about 3Ps individual maturity status to the ‘sequencing and filtering’ section of the results. Please note that maturity data was not available for many samples from the ‘reference populations’ that were originally collected for previous studies. The ‘Conclusions and future directions’ section is now retitled ‘conclusions, limitations, and future directions’ where the use of juveniles and non-spawning adults is discussed.

L268-270: For the 52 3Ps individuals with available maturity data, 53% were immature, 27% were mature but not spawning, 10% were spawning, and 10% were spent.

L454-459: We also note that our study contained more 3Ps juveniles and non-spawning adults than spawning adults, samples with a lack of maturity information, as well as samples from different seasons and years. This combination of samples across maturity stages and time limits our ability to confidently determine each individual’s population of origin, as Northwestern Atlantic cod are known to undertake long migrations [65].

*Line 193: were retained? A verb is missing in this sentence.*

>>>This sentence was incomplete. We added ‘were retained’ to complete it.

L234-237: Post filtering, 52 individuals from 3Ps, 86 individuals from the Northern cod stock complex (29 from 2J, 28 from 3K, 29 from 3L), 136 individuals from Gulf of St. Lawrence cod stocks (64 from 4R, 30 from 4S, 42 from 4T), 12 from the Bay of Fundy (4X), and 33 from the Gulf of Maine (5Y) were retained.

*Line 198: a more conservative approach is to use all outliers. It is well known that different software detects different sets of outliers. How many outliers were detected by each method?*

>>>We added to the results the number of outliers identified by each method. Please see our response about the outlier filtering approach in the related comment above.

L245-247: BayeScan identified 111 outliers, and PCAdapt with the Bonferroni correction identified 25 outliers. All 25 outliers identified by PCAdapt were consensus outliers.

*Line 242: Parts of this should be in the methods.*

>>> We have added the clustering information to the ‘Population structure analyses’ section of the methods.

L253-255: To further investigate the presence of subpopulation clustering, Bay of Fundy (4X), Gulf of Maine (5Y), and Gulf of St. Lawrence (4RST) were removed to see if the clusters remained.

*Line 248: Is exactly half? Would be better to include the percentages.*

>>>Due to the overlapping of PCA clusters, it becomes arbitrary to assign cluster membership for percentages. However, this is not the case for the DAPC, so we have included percentages in the DAPC results sentence to make it clearer.

L310-312: 3Ps had a 54%/46% mix of the clusters which was similar to 3L (48%/52%). The frequency of clusters changes moving north (3K: 25%/75%, 2J: 14%/86%, Fig. 4; Fig. 5).

*Line 375: "Another explanation", I am not sure I understand the difference with the explanation in the previous sentence.*

>>>Agreed, sentence is redundant with explanation above and has been removed.

*Line 378: Complete this paragraph with results from Clucas 2019.*

>>>We have added results from Clucas et al. 2019 to the end of this paragraph.

L434-441: Clucas et al. [22] also found outliers on chromosomes 18 and 21, including Esr2, when comparing cod from St. Pierre Bank (3Ps) to cod in US waters (western Gulf of Maine winter spawners, Georges Bank, Cox Ledge, eastern Gulf of Maine, and Great South Channel). All other outliers they found also corresponded to genes that play a role in various reproductive functions [22]. Due to this, as well as the spatial patterns in these allele frequencies, we speculate that the separation of these clusters may be associated with differences in reproductive function or latitudinal differences in temperature for the northern (Northern cod stock complex Division 2J in particular) and southern stocks (4RST).

*Fig1: Include names of places, people outside Canada might not be familiar with the names.*

>>>We have added the names of places before the stock names in this figure caption.

L177-178: Northern stock complex (southern Labrador to Grand Bank; 2J, 3K, 3L), southern Newfoundland (3Ps), Gulf of St. Lawrence (4R, 4S, 4T), Bay of Fundy (4X), Gulf of Maine (5Y).

*References*

*Clucas, G. V., Lou, R. N., Therkildsen, N. O., & Kovach, A. I. (2019). Novel signals of adaptive genetic variation in northwestern Atlantic cod revealed by whole‐genome sequencing. Evolutionary Applications, 12(10), 1971–1987.* [*https://doi.org/10.1111/eva.12861*](https://doi.org/10.1111/eva.12861)

*Kess, T., Bentzen, P., Lehnert, S. J., Sylvester, E. V. A., Lien, S., Kent, M. P., Sinclair‐Waters, M., Morris, C., Wringe, B., Fairweather, R., & Bradbury, I. R. (2020). Modular chromosome rearrangements reveal parallel and nonparallel adaptation in a marine fish. Ecology and Evolution, 10(2), 638–653.* [*https://doi.org/10.1002/ece3.5828*](https://doi.org/10.1002/ece3.5828)

*Ruzzante, D. E., Taggart, C. T., Lang, S., & Cook, D. (2000). Mixed-Stock Analysis of Atlantic Cod near the Gulf of St. Lawrence Based on Microsatellite DNA. Ecological Applications, 10(4), 1090–1109.* [*https://doi.org/10.2307/2641019*](https://doi.org/10.2307/2641019)

*Therkildsen, N. O., Hemmer-Hansen, J., Als, T. D., Swain, D. P., Morgan, M. J., Trippel, E. A., Palumbi, S. R., Meldrup, D., & Nielsen, E. E. (2013). Microevolution in time and space: SNP analysis of historical DNA reveals dynamic signatures of selection in Atlantic cod. Molecular Ecology, 22(9), 2424–2440.* [*https://doi.org/10.1111/mec.12260*](https://doi.org/10.1111/mec.12260)

**Reviewer 2:**

*The study presents compelling results, emphasizing the importance of incorporating genetic analyses into stock management. Overall, the research is robust, but given the range of analyses conducted, it is essential to provide access to all scripts used for data analysis and figure generation. Additionally, I recommend reviewing the names of the locations to ensure consistency throughout the text and figures, as inconsistencies can make the study difficult to follow.*

>>> We are glad the reviewer finds the results compelling and the research robust. We thank the reviewer for the constructive feedback. All scripts are now available on: <https://github.com/SarahBabaei/3Ps_Atlantic_Cod.>

Specific Comments:

Introduction

*Line 108-111: “Individuals tagged within 3Ps were recaptured during spring and summer on the…”. In the previous sentence, you are referring to several studies and, in this sentence, you mentioned individuals tagged within 3Ps were recaptured but did not say to what study you are referring, which makes the whole paragraph very confusing. In addition, I recommend being more specific instead of using “these studies” in line 109, such as citing them at the end of the sentence.*

>>>We specified the study containing the tagging information. We also added a citation to the end of each sentence to specify which tagging study we refer to.

L106-107: Templeman tagged individuals within 3Ps (St. Pierre Bank) and recaptured them during spring and summer on the Grand Bank (3LNO), Halibut Channel, as well as St. Pierre Bank (3Ps) [31].

Materials and Methods

*Line 132: “Some previously collected samples” How many previously collected samples were included? Please specify this in the text.*

>>> We now specify the exact number.

L163-167: 267 previously collected fin clip and heart muscle samples from juvenile and adult cod were also resequenced for this study, including Northern cod stock complex samples (2J3KL) from spring 2013-2015 published in Puncher et al. [8, 13, 21] (S1 Table) and autumn 2017-2018 samples from annual surveys performed by Fisheries and Oceans Canada and the Atlantic Groundfish Council [8, 21] (2017 samples from Northern cod stock are unpublished).

*Line 138-140: I would include this information in the previous sentence and make it more objective given you are referring to the same set of “previously collected samples”.*

>>>We moved the sentence and included some information in the previous sentence as well.

L163-169: 267 previously collected fin clip and heart muscle samples from juvenile and adult cod were also resequenced for this study, including Northern cod stock complex samples (2J3KL) from spring 2013-2015 published in Puncher et al. [8, 13, 21] (S1 Table) and autumn 2017-2018 samples from annual surveys performed by Fisheries and Oceans Canada and the Atlantic Groundfish Council [8, 21] (2017 samples from Northern cod stock are unpublished). These previously collected samples were caught through acoustic-trawl surveys and industry vessels using bottom trawl gear as outlined in Puncher et al. [8, 13, 21].

*Figure 1 legend: Please add the area that 3Ps represent.*

>>>‘southern Newfoundland (3Ps)’ was added to the legend.

L176-178: Only points from sets represented in the final, filtered full dataset are plotted in red. Northern stock complex (southern Labrador to Grand Bank; 2J, 3K, 3L), southern Newfoundland (3Ps), Gulf of St. Lawrence (4R, 4S, 4T), Bay of Fundy (4X), Gulf of Maine (5Y).

*Line 160: Please mention what happened with the other 350 samples that were not included in the library prep.*

>>>These samples were not included in this study since a lane effect was identified in a preliminary study [48]. We have added this information to the sentence to make this clear.

L200-202: One next generation sequencing library of 384 individuals was prepared using double digest restriction-site associated DNA sequencing (ddRAD). All individuals were sequenced on one lane to avoid lane effects seen in preliminary results [47].

*Line 163: From where did you base the use of these enzymes, did you do any kind of in silico simulation using whole genome?*

>>>For consistency across studies, we used the same enzymes as the ‘reference samples’ originally collected for previous cod population genetic studies that we were comparing 3Ps to.

L202-204: We used the protocol from Puncher et al. [13], a modified protocol from Poland et al. [48] using enzymes PstI and MspI, for consistency with prior ddRAD studies on the reference samples [13, 21].

*Line 187: Please include the acronym alongside the names of the geographic locations. These locations are a key element of the study, as the research focuses on stock identification. Ensure the naming is clear and concise to avoid any confusion for the reader.*

>>>We have added the NAFO Stocks to each population in brackets.

L229-231: Pairwise FST values were calculated for pairs of geographic locations (Northern cod stock complex (2J3KL), Gulf of St. Lawrence (4RST), 3Ps, Bay of Fundy (4X), Gulf of Maine (5Y)) using the R package pegas [55].

*Line 197: Same thing as the comment before, make sure you are concise with the locations name.*

>>>We have removed extra words from the sentence to make locations clearer.

L240-242: Samples were grouped according to their geographic location (Northern cod stock complex (2J3KL), Gulf of St. Lawrence (4RST), southern Newfoundland (3Ps), Bay of Fundy (4X), Gulf of Maine (5Y)).

*Line 215: What multiple methods?*

>>>We agree that this sentence is confusing, we have removed this sentence for clarity.

Results

*Sequencing and filtering: Please reference here the Supplementary table 2 so the reader can understand how you ended up with the given number of neutral SNPs.*

>>>We have added a reference to supplementary table 2 at end of the sequencing and filtering paragraph.

*Line 234-236: Between what populations? This is not clear here nor in the methods.*

>>>We have added ‘all’ to the sentence to make it clear that all population pairs were tested. We have added this to the methods section as well.

L231-233: Manhattan plots were generated for the full dataset (all populations) using these FST values and the R package ggplot2 v.3.0.0 [56] and used to determine the location of linkage groups.

L280-282: A Manhattan Plot using the whole dataset identified four chromosome regions driving most of the differentiation between all population pairs: chromosomes 1, 2, 7, and 12 (Fig 2).

*Line 244: As mentioned before, for the readers that are not familiar with these locations, make sure to include the acronym as well for easier visualization using the map you provided.*

>>>We have added NAFO divisions in brackets for each population in this sentence.

L290-293: One cluster primarily had individuals from Gulf of Maine (5Y) and Bay of Fundy (4X), one mostly contained individuals from the Gulf of St. Lawrence (4RST), the Northern cod stock complex (2J3KL), and 3Ps, and another contained primarily individuals from the Northern cod stock complex (2J3KL) and 3Ps (Fig 3AB).

*Figure 3: Given that your study has multiple locations, you need to make sure the reader does not get confused with the names/acronyms. In figure 3A you used the acronyms and figure 3B the names, which makes extremely confusing to understand and compare the genetic clusters. The same issue is present throughout the text.*

>>> We agree and have made changes to Figure 3’s caption and throughout the manuscript to improve clarity and consistency in naming.

*Table 2: Are these Fst values significant? Please display the p-value somehow.*

>>>Large SNP-based datasets are often too computationally intensive to calculate p-values, and so available R packages use bootstrapped confidence intervals instead. Many large SNP-based studies have used these packages including other cod studies such as:

Clucas, G. V., Kerr, L. A., Cadrin, S. X., Zemeckis, D. R., Sherwood, G. D., Goethel, D., ... & Kovach, A. I. (2019). Adaptive genetic variation underlies biocomplexity of Atlantic Cod in the Gulf of Maine and on Georges Bank. PLoS One, 14(5), e0216992.

Fairweather, R., Bradbury, I. R., Helyar, S. J., de Bruyn, M., Therkildsen, N. O., Bentzen, P., ... & Carvalho, G. R. (2018). Range‐wide genomic data synthesis reveals transatlantic vicariance and secondary contact in Atlantic cod. Ecology and evolution, 8(23), 12140-12152.

*Line 291-294: Please also provide p-values for a more comprehensive interpretation of your results, specially considering the fine scale genetic structure you are referring here.*

>>>Please see previous comment.

Discussion

*Line 375-376: Did the study include perhaps any gene flow or effective migration rate analyses? If not, provide a citation in this sentence that support your affirmation.*

>>>We have added citations to Puncher et al. 2019 and 2020 at the end of this sentence.

*Line 378-386: This is a very interesting finding. Is there any reference to support such findings? Perhaps in another species with similar characteristics/distribution?*

>>>Yes, another study on Atlantic cod found this as well. Clucas et al. 2019 is now discussed in this paragraph.

L435-441: Clucas et al. [22] also found outliers on chromosomes 18 and 21, including Esr2, when comparing cod from St. Pierre Bank (3Ps) to cod in US waters (western Gulf of Maine winter spawners, Georges Bank, Cox Ledge, eastern Gulf of Maine, and Great South Channel). All other outliers they found also corresponded to genes that play a role in various reproductive functions [22]. Due to this, as well as the spatial patterns in these allele frequencies, we speculate that the separation of these clusters may be associated with differences in reproductive function or latitudinal differences in temperature for the northern (Northern cod stock complex Division 2J in particular) and southern stocks (4RST).
