# Supplementary material for: Genome-wide SNPs reveal novel genetic relationships among Atlantic cod (Gadus morhua) from the south coast of Newfoundland, Canada (subdivision 3Ps), Northern cod stock complex, and Gulf of St Lawrence
Source: PLoS One. 2025 Mar 14;20(3):e0317768. doi: 10.1371/journal.pone.0317768 (PMC11908700; doi:10.1371/journal.pone.0317768)
Supplement: Attachment — Submitted filename: Response_to_Reviewers_auresp_2.docx [file pone.0317768.s014.docx]

**Reviewer #1***:*

*I reviewed the first version of the manuscript. This version is an improved version and it is clear the authors have made an important effort to attend to both reviewer's comments. I just have one recommendation:*

*I don't see mention of using the 3rd method (Benjamini-Hochberg) for outlier detection in the methods, I think would be important to add a sentence and add the results removing all outliers in the supplementary material as the authors have already done the analysis.*

>>>We are glad the reviewer finds this manuscript version improved and we would like to thank them for their recommendation. We have now included a description of the third outlier detection method (Benjamini-Hochberg) in the methods and included the results from removing all outliers from all outlier detection methods in the Supplementary Information (S1 Fig.).

L247-250: To explore the use of a third outlier detection method, we used PCAdapt with a Benjamini-Hochberg correction. This returned 67 outliers but left the consensus outliers unchanged. See S1 Fig. for PCA clustering results involving removal of all outliers from all three outlier detection methods.

L697-701: **S1 Figure. PCA with all outliers removed from all three outlier detection methods.** Detection methods include BayeScan, PCAdapt with Bonferroni correction, and PCAdapt with Benjamini-Hochberg correction. Consensus outliers remained the same with addition of third outlier detection method (25 consensus). Northern = Northern cod stock (2J3KL), GSL = Gulf of St. Lawrence (4RST), BoF = Bay of Fundy (4X), GoM = Gulf of Maine (5Y).
